# Lathyrane Diterpenoids: Half-Century of Chemical Insights and Therapeutic Potential

**DOI:** 10.3390/plants15142235

**Published:** 2026-07-22

**Authors:** Diego Caprioglio, Daniela Imperio, Alberto Minassi

**Affiliations:** 1 Department of Pharmaceutical Science, University of Piemonte Orientale, Via Bovio 6, 28100 Novara, Italy; 2 Department for Sustainable Development and Ecological Transition, University of Piemonte Orientale, P.zza St. Eusebio 5, 13100 Vercelli, Italy; daniela.imperio@uniupo.it

**Keywords:** natural products, secondary metabolites, lathyrane diterpenoids, transannular rearrangements, bioactive compounds

## Abstract

Despite being discovered over a century ago, lathyrane diterpenoids have only recently attracted significant attention from the organic chemistry community due to their distinctive carbon framework, characterized by a 5/11/3 tricyclic system. The conformational rigidity of these scaffolds promotes close spatial proximity between functional groups, making transannular reactions a powerful tool for the construction of new complex polycyclic architectures. In parallel, compounds from the genus *Euphorbia* have emerged as versatile molecular frameworks in medicinal chemistry, exhibiting a wide range of biological activities, including cytotoxic, antiviral, and neuroprotective effects. This review summarizes recent advances in the field, with particular emphasis on synthetic strategies, structural diversity, and medicinal chemistry aspects of this intriguing class of secondary metabolites.

## 1. Introduction

Secondary metabolites have long fascinated both chemists and pharmacologists due to their structural complexity and intriguing pharmacological profiles [1,2]. Among the various classes of natural products, terpenes constitute the largest and most structurally diverse family. This vast structural complexity derives from very simple biosynthetic roots, characterized by a cyclase phase in which cationic polyene cyclizations produce a myriad of products typically containing multiple fused rings and several stereocenters [3]. The high structural complexity of terpenes underpins their ability to interact with diverse biological targets, to the extent that some of these frameworks can be defined as “*privileged scaffolds*” from a medicinal chemistry perspective. Their ability to perturb different molecular targets is likely the result of evolutionary adaptation, which allowed living organisms to develop “*functional molecules*” for antagonistic, antifeedant, and ecological interaction purposes [4].

**Scheme 1:**
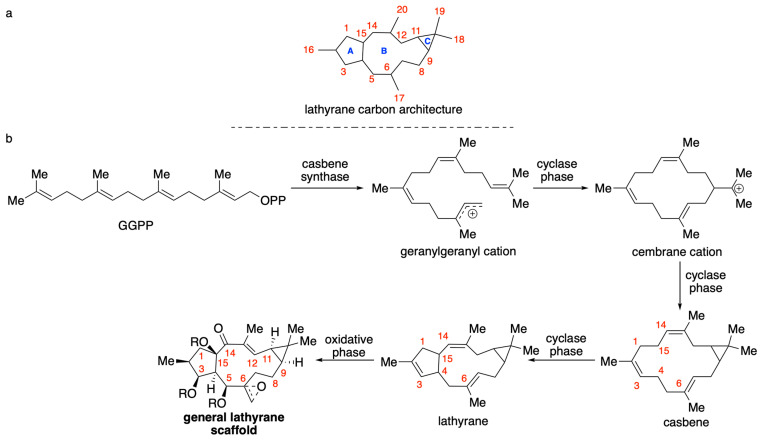
(**a**) General carbon architecture of lathiranes, with no stereochemical detail. Carbocyclic rings are labeled with the letters A, B and C; (**b**) proposed biosynthetic pathway of lathyrane diterpenoids.

Lathyrane diterpenoids are members of this large terpene family, characterized by a twenty-carbon skeleton organized into a 5/11/3 tricyclic system commonly decorated with various oxygenated functions (Scheme 1a). They represent the primary chemical components of the genus *Euphorbia*, which comprises more than 2000 recognized species widely used in various traditional medicines [5]. Although the first lathyrane was discovered by Tahara in 1890 [6], this class of secondary metabolites has only truly attracted the attention of the scientific community in the present century, leading to the isolation of numerous derivatives from diverse sources. From a biosynthetic standpoint, it is generally hypothesized that they arise from the electrophilic cyclization of geranylgeranyl pyrophosphate (GGPP) via an allyl cation intermediate, catalyzed by casbene synthase. The cyclase phase begins with the generation of the cembrane cation, which is eventually transformed into the lathyrane scaffold—first through the installation of the cyclopropane ring, and subsequently via bond formation between C4 and C15 [7,8]. Once assembled, the polycyclic architecture undergoes an oxidative phase, during which the terpene skeleton is oxidatively functionalized. This includes the insertion of hydroxyl groups (mainly at positions C3, C5, and C15), the introduction of a ketone moiety at C14, a conjugated endocyclic *trans*-double bond, and an exocyclic double bond at C6, which is epoxidized in some derivatives. Moreover, structural complexity is further increased by the esterification of these hydroxyl groups with both aliphatic and aromatic acids (Scheme 1b).

Owing to its intrinsic rigidity and dense functionalization, the tricyclic system of lathyrane diterpenoids seems to have been tailored by nature to interact with diverse biological systems. Many natural derivatives have been evaluated against various biological targets, with several demonstrating cytotoxic, anti-inflammatory, and antiviral activities [9,10]. Their MDR has been extensively studied, highlighting the ability of this compound class to modulate P-glycoprotein activity in various tumor cell lines [5]. Furthermore, the capacity of certain lathyranes to stimulate NPC proliferation has recently emerged in both in vitro and in vivo assays, opening new avenues for the development of therapeutics targeting diseases associated with impaired neurogenesis [11].

While the biological aspects of natural lathyranes have been extensively reviewed [5,9,10,12], a comprehensive overview of their chemical space and medicinal chemistry applications is, to the best of our knowledge, still lacking. In light of the growing interest in this class of secondary metabolites, this highlight aims to systematically cover the literature regarding the chemical transformations and skeletal rearrangements of natural lathyranes, as well as the medicinal applications and biological activities of their semisynthetic derivatives. The manuscript is organized by first addressing the reactivity of the tricyclic scaffold—classified according to the mode of activation (acidic, photochemical, or basic conditions)—and subsequently focusing on the medicinal chemistry aspects.

## 2. Chemistry of Lathyranes

The ambition to replicate the efficiency and selectivity of natural processes under laboratory conditions—in a sort of “*cyclase phase in a flask*”—has inspired the organic chemistry community to explore the chemical space and the reactivity of the lathyrane scaffold. This effort has resulted in the discovery of novel rearrangements, unprecedented carbon architectures, and new biomimetic synthetic strategies. In this section, we report all the published articles with an in-depth analysis of the conditions, mechanisms and outputs of the reactions.

### 2.1. Acid-Mediated Reactions

**Figure 1:**
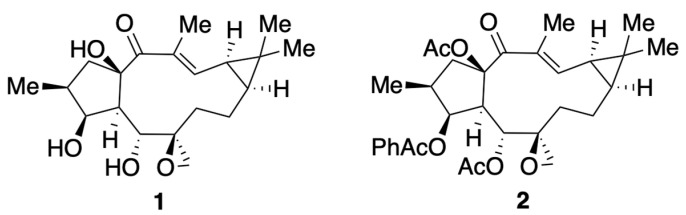
Structures of epoxylathyrol (**1**) and euphorbia factor L_1_ (**2**).

The first member of this class—originally referred to by the archaic name “euphorbiasteroids”—was isolated as early as 1890 [6]. However, it was not until 1970 that a pair of landmark papers were published, reporting the X-ray analysis and complete structural elucidation of two new compounds: epoxylathyrol (**1**) and euphorbia factor $L_{1}$ (**2**) (Figure 1) [13]. In their attempt to determine the position of the functional groups present on the diterpene skeleton, the authors described the very first transannular cyclization of this tricyclic system.

**Scheme 2:**
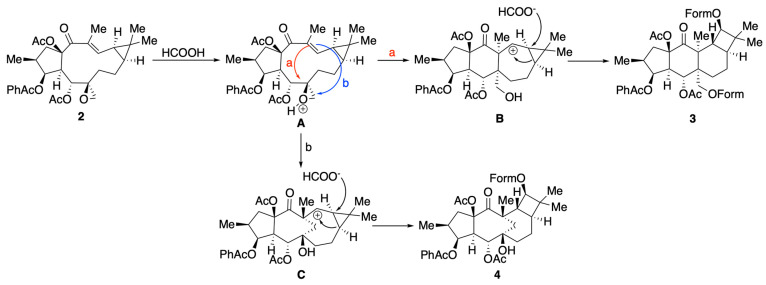
Mechanisms of acid-catalyzed cyclization of euphorbia factor L_1_ (**2**) with the production of compounds **3** (red path a) and **4** (blue path b).

Treatment of compound **2** with formic acid yielded two tetracyclic products, **3** and **4**, in which the cyclopropane ring was replaced by a cyclobutane ring, while the macrocyclic system was transformed into [4.4.0] and [4.4.1] bicyclic frameworks, respectively. The reaction proceeds in an identical manner in other acidic media, such as ${\mathrm{AcOH}/\mathrm{HClO}}_{4}$ and ${\mathrm{CF}}_{3}\mathrm{COOH}$. Compounds **3** and **4** are proposed to derive from an intramolecular alkylation of C13 at both termini of the protonated epoxide (**A**), followed by a ring expansion of the resulting cyclopropylcarbinyl carbocations (**B** and **C**) (Scheme 2) [13].

**Figure 2:**
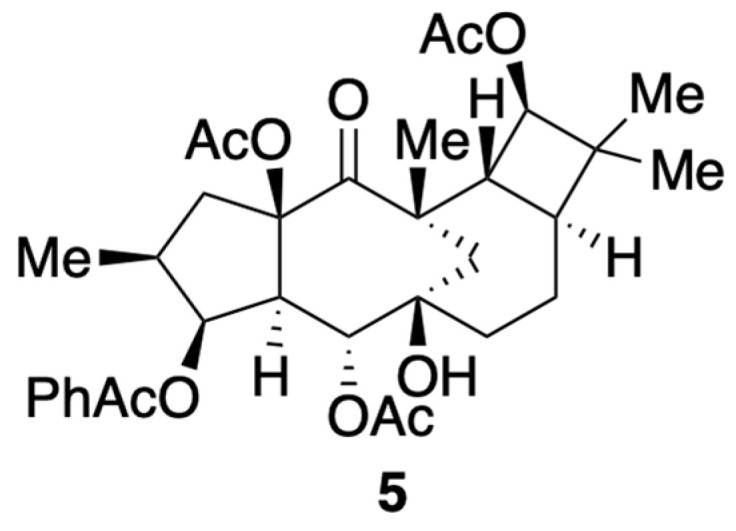
Structure of compound **5**.

In 1973, Takemoto reported a transannular cyclization induced by the exposure of euphorbia factor $L_{1}$ (**2**) to an acidic system composed of oxalic acid in glacial acetic acid. The rearranged product **5** shares the same tetracyclic framework as compound **4**, with the replacement of the formyloxy moiety at C11 with an acetoxy group (Figure 2) [14].

**Scheme 3:**
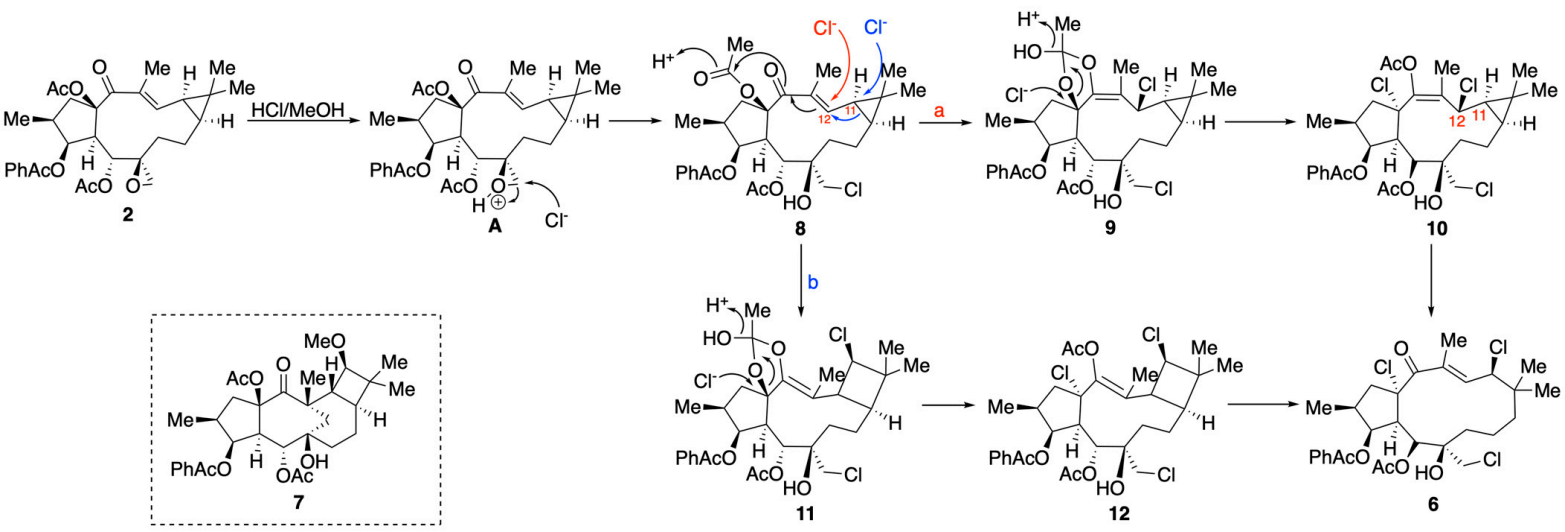
Mechanism of acid-catalyzed cyclization of euphorbia factor L_1_ (**2**) with the production of compounds **6** and **7**.

Two years later, the treatment of a methanolic solution of euphorbia factor $L_{1}$ (**2**) (erroneously named epoxylathyrol **1** in the original article) with an excess of mineral acids (HCl, $H_{2}{\mathrm{SO}}_{4}$) was reported to afford two distinct rearranged products, **6** and **7**, respectively [15]. While compound **7** shares the same tetracyclic core as **4** featuring a methoxy moiety in place of the formyloxy group, the trichlorinated derivative **6** (obtained in 45% yield) is characterized by an unprecedented 5/12 bicyclic system incorporating three chlorine atoms. The proposed mechanism for the formation of **6** starts with the protonated epoxide **A**, which undergoes ring-opening via chloride attack to afford the corresponding halohydrin **8**. According to the authors, intermediate **8** can diverge into two possible pathways (Scheme 3). In the first pathway (red route), the insertion of the second chlorine atom occurs via a Michael-type addition at C12, generating intermediate **9**. Upon protonation, **9** undergoes a third nucleophilic attack by chloride to yield compound **10**. Deacetylation, followed by a chlorine migration from C12 to C11 and the concomitant cleavage of the cyclopropane ring, ultimately delivers the final product **6.** Alternatively, the second pathway (blue route) involves a chloride attack at C11 of **8**, promoting a concerted transannular cyclization to form the cyclobutane-containing intermediate **11**. Subsequent protonation and substitution of the acetyl group by the third chloride ion yields intermediate **12**, which eventually evolves into the final product **6** [15].

**Scheme 4:**
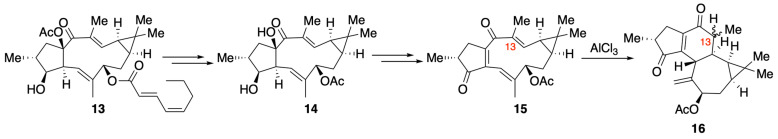
Synthesis of compound **16** from euphohelioscopin A (**13**).

In 1984, Yamamura and co-workers described an elegant biomimetic approach for the interconversion of the lathyrane scaffold into the jatropholane skeleton [16]. Jatropholane-type diterpenes represent a rare class of secondary metabolites characterized by a unique 5/6/7/3 tetracyclic ring system, found in several plants belonging to the genus *Euphorbia*. In order to mimic the biosynthetic cascade, compound **14** (derived from euphohelioscopin A, **13**) was manipulated to afford the diketo-derivative **15**. Upon exposure to ${\mathrm{AlCl}}_{3}$, **15** underwent a transannular cyclization via the formation of a new carbon–carbon bond between C5 and C12, leading to a mixture of C13-epimers (**16**) as the major products in 45% yield (Scheme 4).

**Figure 3:**
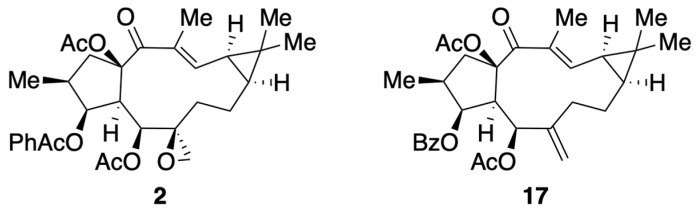
Structures of compounds **2** and **17**.

While the pioneering studies described above were largely limited to a preliminary exploration of lathyrane reactivity, at the turn of the century, several research groups began to investigate the chemical behavior of these secondary metabolites more systematically. A seminal paper in this context was published in 2001 by Appendino and co-workers, who detailed the diverse skeletal rearrangements induced by both Brønsted and Lewis acids on euphorbia factors $L_{1}$ (**2**) and $L_{3}$ (**17**) [17]. These two natural congeners share the identical diterpene core, and their primary structural divergence lies in the nature of the C6–C17 connectivity: in $L_{1}$ (**2**) the two carbons are incorporated into an epoxide ring, whereas in $L_{3}$ (**17**) they are involved in an olefinic double bond. A secondary difference, negligible from a reactivity standpoint, resides in the esterification profile at C3, with **2** bearing a phenylacetate ester and **17** featuring a benzoate moiety (Figure 3).

**Scheme 5:**
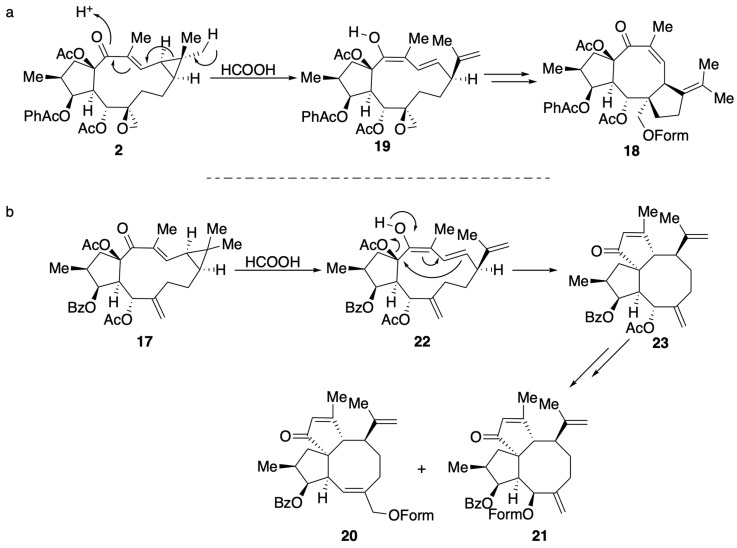
(**a**) Mechanism of acid-catalyzed cyclization of euphorbia factor L_1_ (**2**) with the production of compound **18**; (**b**) mechanism of acid-catalyzed cyclization of euphorbia factor L_3_ (**17**) with the production of compounds **20** and **21**.

The authors decided to use formic acid and Yb(OTf)_3_ to exploit the captodative nature of the endocyclic alkene. In formic acid, euphorbia factor $L_{1}$ (**2**) confirmed its expected reactivity, yielding compounds **3** (42%) and **4** (33%) as the major products, alongside the cyclooctanoid derivative **18** as a minor component (6%) (Scheme 5a). Although compounds **3** and **4** were already known, the authors noted that the previously assigned stereochemistry at the bridgehead positions of the central bicyclic core was incorrect, and they consequently performed a complete revision of their spatial configuration. Conversely, $L_{3}$ (**17**) exhibited a completely different behavior under identical conditions, giving rise to compounds **20** and **21**. The proposed mechanism involves the cleavage of the cyclopropane moiety, which generates intermediate **23** via the formation of a new carbon–carbon bond between C11 and C15, accompanied by the extrusion of an acetyl group. Finally, this intermediate underwent solvolysis, replacing the C5-acetate with a formate group, to deliver the isomeric products **20** (19%) and **21** (11%) (Scheme 5b) [17].

**Scheme 6:**
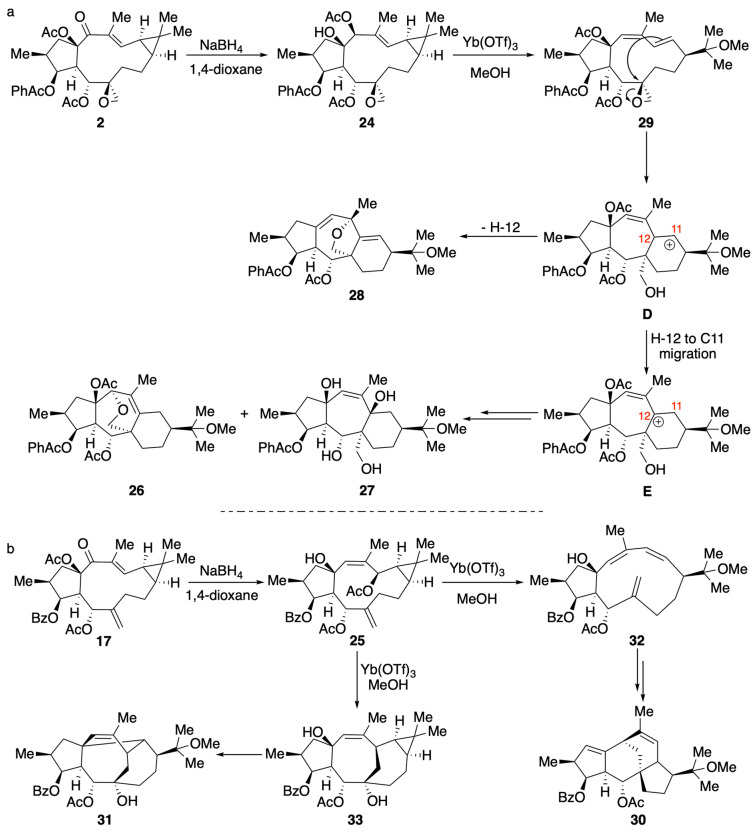
(**a**) Mechanism of acid-catalyzed cyclization of euphorbia factor L_1_ (**2**) with the production of compounds **26**, **27** and **28**; (**b**) mechanism of acid-catalyzed cyclization of euphorbia factor L_3_ (**17**) with the production of compounds **30** and **31**.

In order to enhance the nucleophilicity of the endocyclic double bond and explore novel rearrangement pathways, the authors reduced the enone carbonyl of both $L_{1}$ (**2**) and $L_{3}$ (**17**), obtaining the corresponding alcohol derivatives **24** and **25**. These intermediates were then exposed to Lewis acid conditions using ${\mathrm{Yb}(\mathrm{OTf})}_{3}$ in MeOH. Compound **24** underwent a complete skeletal rearrangement, interconverting the lathyrane architecture into the *abeo*-mirsane framework, affording compounds **26** (21%), **27** (19%), and **28** (35%). The latter are proposed to derive from the rearrangement of a common intermediate characterized by a *C-seco*-lathyradiene motif (**29**), which undergoes a transannular cyclization triggered by the *exo*-opening of the epoxide ring to generate carbocation **D**. Following a series of hydride shifts, the positive charge is eventually quenched via intermolecular trapping by the oxygenated solvent, delivering the mixture of final products (Scheme 6a).

On the other hand, compound **25** under identical conditions led to the formation of two major products, **30** (22%) and **31** (15%), arising from two proposed key intermediates, **32** and **33**, respectively. The triene **32**, favored by the spatial proximity of the alkenes, is hypothesized to undergo a concerted, Diels–Alder-type cyclization to furnish the complex tetracyclic core of compound **30**. Conversely, intermediate **33** undergoes the loss of a water molecule promoted by the nucleophilic attack of a molecule of MeOH at C10, yielding the final compound **31** (Scheme 6b) [17].

**Scheme 7:**
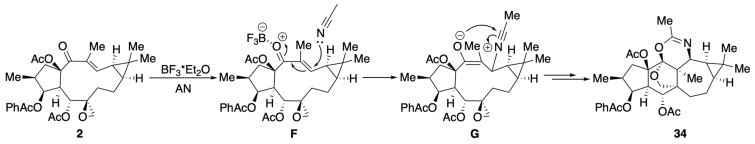
Mechanism of acid-catalyzed cyclization of euphorbia factor L_1_ (**2**) with the production of compound **34**.

In 2019 and 2020, Zhou and co-workers published two comprehensive studies describing the reactivity of euphorbia factor $L_{1}$ (**2**) in the presence of BF_3_*Et_2_O as a catalyst in non-protic solvents, such as ethyl acetate (EtOAc) and acetonitrile (MeCN) [18,19]. In both papers, they successfully interconverted the lathyrane scaffold into the euphoractane and myrsinane frameworks; furthermore, they uncovered a novel, unnatural 5/7/7/4 fused-ring diterpene skeleton. Interestingly, while EtOAc acted primarily as a nucleophilic source of an acetate moiety incorporated into the final rearranged products [18], AN was able to actively participate in the reaction by interacting with the activated enone system, leading to an unprecedented euphoractine B-type pseudo-alkaloid (**34**). To account for the formation of this nitrogen-containing derivative, the authors suggested a mechanism wherein the ${\mathrm{BF}}_{3}$-activated intermediate **F** undergoes a Michael-type attack by a molecule of MeCN at C12, generating the zwitterionic species **G**, which eventually evolves into the final pseudo-alkaloid **34** (Scheme 7) [19].

**Scheme 8:**
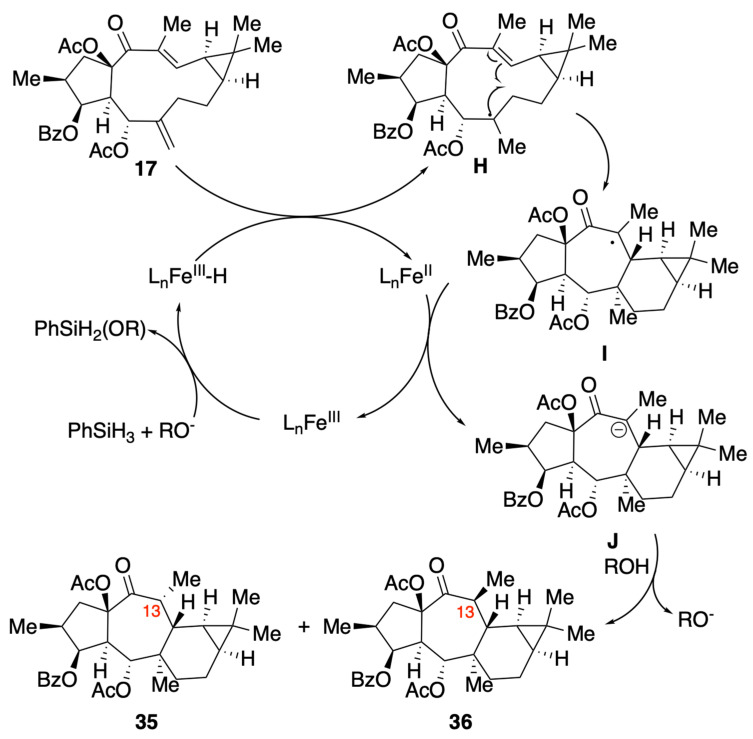
Mechanism of radical-reductive cyclization of euphorbia factor L_3_ (**17**) with the production of compounds **35** and **36**.

In 2021, Gao and co-workers investigated the biogenetic relationship between lathyranes and premyrsinanes, two structurally related diterpenoid skeletons commonly isolated from *Euphorbia* species. To probe whether these frameworks were biosynthetically interconnected, the authors subjected euphorbia factor $L_{3}$ (**17**) to radical reductive conditions, employing ${\mathrm{PhSiH}}_{3}$ as a hydrogen donor and ${\mathrm{Fe}(\mathrm{acac})}_{3}$ as a radical mediator [20]. The reaction furnished a C13-epimeric mixture of compounds **35** and **36**, in which the lathyrane architecture was effectively reconfigured into the premyrsinane core via a radical-mediated skeletal rearrangement. The proposed mechanism initiates with the formation of an iron(III) hydride species, which undergoes a hydrogen atom transfer (HAT) to generate the C6 radical intermediate **H**. This species then participates in a C6–C12 cyclization to afford the radical intermediate **I**, successfully installing the 5/7/6/3 fused tetracyclic system. A subsequent single-electron transfer (SET) oxidation furnishes the cationic intermediate **J**, which is eventually quenched by proton abstraction from the reaction medium to yield the final products (Scheme 8).

**Scheme 9:**
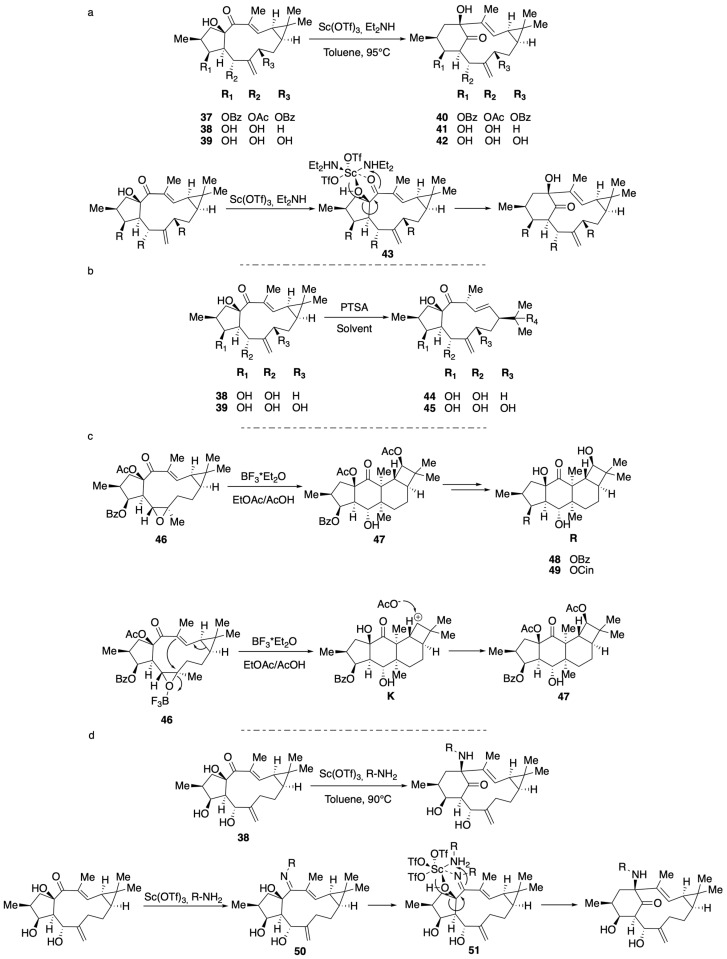
(**a**) Mechanism of acid-catalyzed cyclization of compounds **37**–**39** with the production of compounds **40**–**42**; (**b**) mechanism of acid-catalyzed cyclization of compounds **38** and **39** with the production of compounds **44** and **45**; (**c**) mechanism of acid-catalyzed cyclization of compound **46** with the production of compounds **47**–**49**; (**d**) mechanism of acid-catalyzed cyclization of compound **38** with the production of lathyranone alkaloids.

Following their initial study, Gao and co-workers initiated a systematic investigation into the reactivity of the lathyrane scaffold, aiming to develop new biomimetic strategies to reorganize this 5/11/3 tricyclic system into other natural diterpene skeletons. In the first study of this series, a small set of lathyranes (**37**–**39**) was treated with catalytic amounts (10 mol% each) of ${\mathrm{Sc}(\mathrm{OTf})}_{3}$ and ${\mathrm{Et}}_{2}\mathrm{NH}$, affording lathyranones (**40**–**42**) in excellent yields (≈90%) on a milligram scale [21]. The authors postulated that the formation of a metal–chelate complex (**43**) was the key intermediate favoring the conversion of the starting material into the lathyrone scaffold. Upon coordination of ${\mathrm{Sc}(\mathrm{OTf})}_{3}$ to the carbonyl and the neighboring C15 hydroxyl group, an α-ketol rearrangement took place, driving the migration of the C1–C15 bond to the C1–C14 position (Scheme 9a).

Following the success obtained with ${\mathrm{Sc}(\mathrm{OTf})}_{3}$, the authors explored the potential of p-toluenesulfonic acid (PTSA) in promoting structural rearrangements of lathyrol (**38**) and 7-hydroxylathyrol (**39**). Under optimized conditions, both substrates underwent selective and complete conversion into the corresponding 10,11-*seco*-derivatives [22]. Notably, 10,11-*seco*-lathyrane diterpenes constitute a small and rare group of secondary metabolites isolated from *Jatropha* species, characterized by a distinctive and uncommon bicyclo [9.3.0] tetradecane skeleton. The extreme rarity of these diterpenes (only five isolated derivatives known), combined with the typical acidity of the soil in which these plants grow, led the authors to speculate that this small group of molecules might actually represent artifacts formed during the storage and extraction processes of the plant material. In support of this hypothesis, heating **38** and **39** to 60 °C in the presence of PTSA confirmed that Brønsted acids exert a powerful influence on product formation (Scheme 9b). The authors characterized a small library of variously functionalized *seco*-derivatives, which are proposed to derive from carbonyl protonation followed by epoxide ring-opening to yield a tertiary carbocation, subsequently trapped by the solvent.

Inspired by Zhou’s previous work, the same research group explored the reactivity of euphorbia factor $L_{12}$ (**46**) in the presence of BF_3_*Et_2_O [23]. The presence of the epoxide ring embedded within the macrocyclic system proved crucial, allowing the identification of a highly efficient and selective pathway to reorganize the lathyrane skeleton into the euphoractane framework. Specifically, the use of BF_3_*Et_2_O in combination with AcOH resulted in the exclusive formation of the euphoractine A skeleton (**47**) in 94% yield. This approach proved sufficiently robust and tunable to allow the gram-scale synthesis of euphoractines M (**48**) and N (**49**) in high yields (Scheme 9c). In parallel, an alternative approach was explored to synthesize a new class of lathyranone alkaloids by exploiting the potential of the Voigt condensation [24]. In this reaction, originally discovered in 1886, an α-hydroxy ketone reacts with a primary or secondary amine under acidic conditions to deliver an α-amino ketone as the primary product [25]. Under optimized conditions, lathyrol (**38**) was heated to 90 °C in toluene in the presence of ${\mathrm{Sc}(\mathrm{OTf})}_{3}$, and a primary amine as the nitrogen source. This strategy achieved the incorporation of a nitrogen atom and the remodeling of the carbon skeleton in a single step (Scheme 9d) [24].

**Scheme 10:**
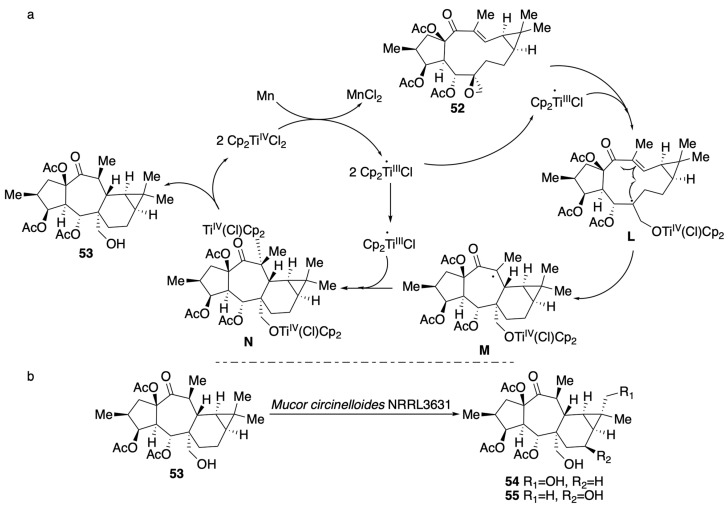
(**a**) Mechanism of titanium-catalyzed cyclization of compound **52** with the production of compound **53**; (**b**) microbial hydroxylation of compound **53** with the production of compounds **54** and **55**.

In 2024, Duran-Patrón and co-workers, inspired by the biogenetic relationship between 6,17-epoxylathyranes and premyrsinanes originally proposed by Appendino’s group, described a new biomimetic approach based on the action of ${\mathrm{Cp}}_{2}{\mathrm{Ti}}^{\mathrm{III}}\mathrm{Cl}$ as a radical mediator [26]. The latter has emerged as a versatile reagent for inducing SET reactions, particularly due to its ability to open epoxide moieties via homolytic cleavage to generate β-titanoxyl radicals [27,28,29]. Starting from the readily available lathyrane derivative epoxyboetirane A (**52**), the authors successfully installed the 5/7/6/3 tetracyclic system typical of premyrsinanes, obtaining compound **53** in very good yield (79%). The proposed mechanism initiates with the generation of ${\mathrm{Cp}}_{2}{\mathrm{Ti}}^{\mathrm{III}}\mathrm{Cl}$ via the reduction of ${\mathrm{Cp}}_{2}{\mathrm{Ti}}^{\mathrm{IV}}{\mathrm{Cl}}_{2}$ with Mn powder. The resulting catalyst reacts with the epoxide of **52** to produce the corresponding β-titanoxyl radical **L**, which undergoes a transannular cyclization to generate the carbon-centered radical **M**. This intermediate is then trapped by a second equivalent of ${\mathrm{Cp}}_{2}{\mathrm{Ti}}^{\mathrm{III}}\mathrm{Cl}$, leading to the alkyltitanium species **N**. The final product **53** arises from the reaction of intermediate **N** with 2,4,6-trimethylsilylpyridinium chloride, thereby regenerating ${\mathrm{Cp}}_{2}{\mathrm{Ti}}^{\mathrm{IV}}{\mathrm{Cl}}_{2}$ (Scheme 10a). To introduce further structural complexity, the authors subjected the premyrsinane-type derivative **53** to biotransformation using *Mucor circinelloides* NRRL3631, a strain known for its ability to metabolize macrocyclic diterpene skeletons, successfully obtaining two new hydroxylated derivatives (**54** and **55**) in moderate yields (Scheme 10b) [26].

### 2.2. Photochemical Reactions

To trace the earliest studies on the photoreactivity of the lathyrane skeleton, it is necessary to go back to 1971, when Ourisson identified the compounds formed upon irradiation of epoxylathyrol (**1**) and $L_{1}$ (**2**) under UV light (254 nm) [30]. The main product was a *seco*-furan (**56**) arising from the generation of a resonance-stabilized carbene intermediate (**P**), which was produced through the *trans*–*cis* isomerization of the conjugated double bond followed by the cleavage of the C10–C11 bond. Eventually, this key intermediate underwent cyclization via attack on the carbonyl oxygen to afford the final furan derivative **56** in quantitative yield.

**Scheme 11:**
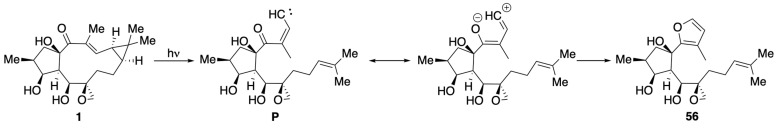
Mechanism of photoinduced synthesis of compound **56** from epoxylathyrol (**1**).

In 1975, another group of French researchers confirmed the results disclosed by Ourisson, not only providing a full characterization of the obtained *lumi*-products, but also thoroughly investigating the reaction mechanism, thereby confirming carbene **P** as the pivotal intermediate in the formation of the final product (Scheme 11) [31].

After these pioneering studies, the photochemistry of lathyranes was largely neglected by the organic chemistry community for nearly forty-seven years, until the advent of modern photocatalysis. In 2022, Gao and co-workers investigated the reactivity of $L_{2}$ (**57**) under photocatalytic conditions using an iridium catalyst under blue and purple LED irradiation, disclosing an efficient biomimetic route to access a class of lathyranes characterized by a *trans*-cyclopropane moiety [32]. Some members of this rare group of secondary metabolites were originally isolated by Ghisalberti [33] and later by Chen [34], who proposed a light-induced process involving the activation of the enone system followed by the isomerization of the cyclopropane moiety as a possible biogenetic pathway.

**Scheme 12:**
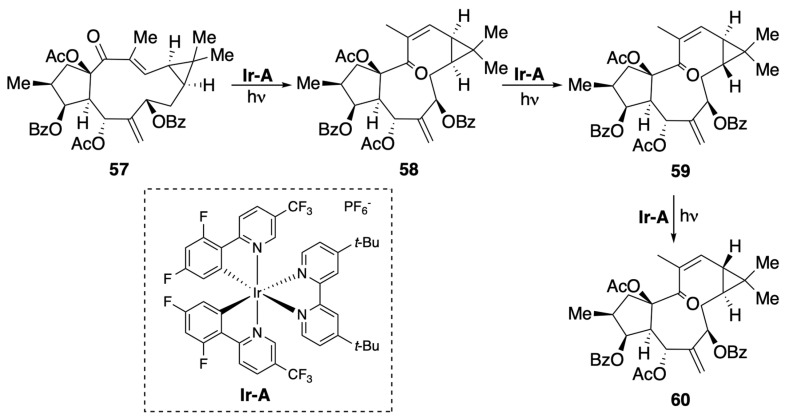
Photoinduced interconversion of L_2_ (**53**) into L_2a_ (**55**) and synthesis of compound **56**.

To explore this hypothesis, the authors exposed $L_{2}$ (**57**) to blue LEDs, which promoted the isomerization of the enone system to yield the corresponding *cis*-isomer **58**. This intermediate underwent a subsequent isomerization of the cyclopropane ring with an inversion of configuration at C9, affording the natural *trans*-isomer $L_{2a}$ (**59**). These results strongly supported the previous hypotheses regarding the key role played by solar radiation in the biogenetic pathway of these compounds. Upon prolonged irradiation (12 h) with purple LEDs, compound **59** eventually furnished a new *trans*-derivative, **60**, featuring an inversion of configuration at both C9 and C11. The authors proposed that the initial photoisomerization of the enone system significantly increases the ring strain of the macrocycle, which is subsequently released through the stepwise isomerization of the cyclopropane moiety, acting as the driving force for the entire rearrangement (Scheme 12).

The exploration of chemical space within a specific class of compounds can lead not only to the discovery of unprecedented rearrangements, but also to the strategic decoration of the starting framework with novel functional groups. In a continuation of their studies on lathyrane reactivity, Gao and co-workers exploited the unique flexibility of this terpenic system toward transannular cyclizations to synthesize premyrsinane and myrsinane derivatives incorporating an aromatic thioether functionality [35].

More specifically, exposure of euphorbia factor $L_{3}$ (**17**) to an equimolar amount of thiophenol under blue LED irradiation led to compound **61**, which features a premyrsinane scaffold bearing a thioether group at C17. Subsequent exposure of **61** to purple LEDs in the presence of a catalytic amount of 4-bromothiophenol (10 mol%) promoted the homolytic cleavage of the cyclopropane ring, generating the corresponding myrsinane thioether **62**.

**Scheme 13:**
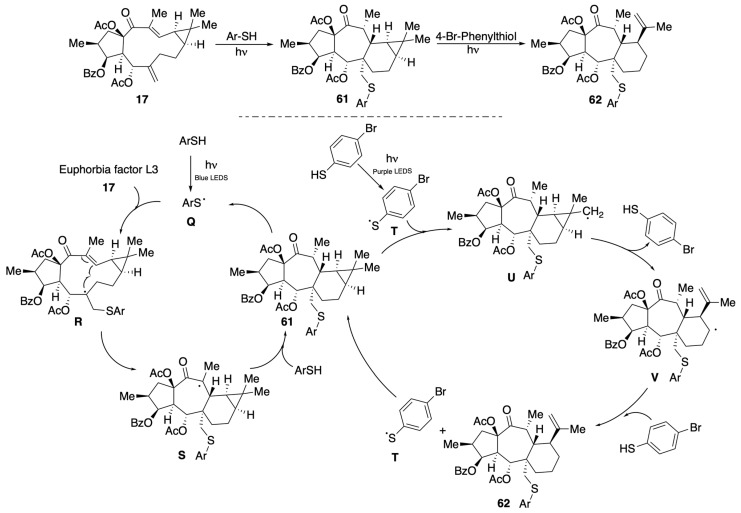
Synthesis of compounds **61** and **62** from euphorbia factor L_3_ (**17**).

The authors proposed a mechanism wherein the thiophenol plays a dual role in both reaction activation and radical propagation. Under light irradiation, the thiol group is activated to generate a reactive thiyl radical (**Q**), which attacks the lathyrane scaffold at C17 to produce the tertiary radical intermediate **R**. This species undergoes a rapid transannular cyclization, establishing a new C6–C12 bond and yielding the tertiary carbon radical **S**, which is eventually quenched via hydrogen atom abstraction from another molecule of thiophenol. This final step of the cascade was elegantly confirmed by using deuterated thiophenol (PhSD), which successfully delivered the corresponding C13-deuterated product. The subsequent interconversion of compound **61** into **62** was similarly enabled by thiyl radical activation, driving the opening of the cyclopropane ring to install the myrsinane framework (Scheme 13). The generality of this transformation was demonstrated using a diverse set of aromatic thiols, affording a small library of derivatives in moderate-to-good yields with high diastereoselectivity (*dr*).

Plants of the genus *Euphorbia* produce a wide variety of diterpenes; some are accumulated in considerable amounts, whereas others, such as eupholathones, are present only in trace quantities. Euphornin E (**63**) [36] is a prominent member of this small family of rearranged diterpenes. Characterized by a complex 5/7/7/4 tetracyclic system, it is biosynthetically speculated to derive from a deep remodeling of the classical lathyrane architecture through transannular cascades.

**Scheme 14:**
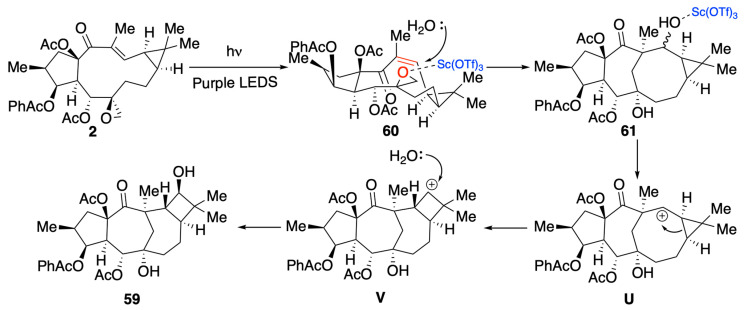
Synthesis of compound **63** from euphorbia factor L_1_ (**2**).

In their ongoing efforts to develop biosynthetically inspired approaches to convert highly abundant lathyrane diterpenes into much rarer congeners, Gao and co-workers recently combined the oxophilic Lewis acidic properties of ${\mathrm{Sc}(\mathrm{OTf})}_{3}$ with the activating effects of photochemistry [37]. This dual strategy enabled the highly efficient, high-yielding conversion of the abundant euphorbia factor $L_{1}$ (**2**) into the rare euphornin E (**63**). Within this reaction pathway, photochemistry plays a crucial role by promoting a rapid and complete *trans*–*cis* isomerization of the enone double bond. This structural change alters the macrocyclic conformation, bringing the epoxide ring into close spatial proximity with the reacting alkene. Subsequently, the resulting *cis*-intermediate **64** undergoes an intramolecular cyclization induced by ${\mathrm{Sc}(\mathrm{OTf})}_{3}$ coordination, successfully installing a transient 5/7/8/3 tetracyclic framework (**65**). The generation of a carbocation at C12 triggers a subsequent Wagner–Meerwein rearrangement, giving rise to the ring-contracted cationic intermediate **Y**, which is eventually trapped by a molecule of water from the medium to afford the final natural product **63** (Scheme 14).

### 2.3. Base-Induced Reactions

Exploration of the reactivity of the tricyclic system of lathyranes has led to the discovery of novel biomimetic approaches toward several classes of secondary metabolites that are scarce in nature. Whereas acid-catalyzed and photochemical transformations have been extensively investigated, the use of basic conditions remains comparatively underexplored, with only limited precedent reported to date.

**Scheme 15:**
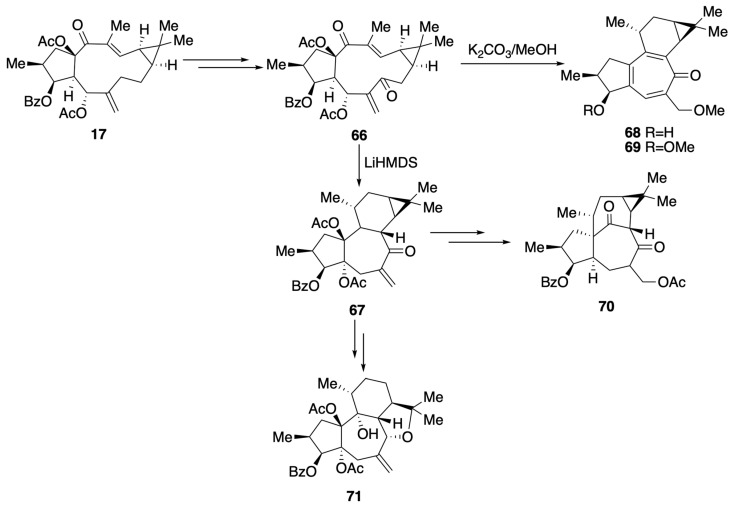
Synthesis of compounds **67**–**71** from euphorbia factor L_3_ (**17**).

Recently, Gao and co-workers demonstrated that treatment of compound **66** (obtained from euphorbia factor $L_{3}$, **17**) under aldol conditions triggers a transannular cyclization, forming a new C8–C14 bond and thereby furnishing the tetracyclic core typical of tigliane diterpenoids [38]. Notably, the nature of the base plays a decisive role in dictating the reaction outcome: treatment with LiHMDS affords compound **67** in 65% yield, whereas the use of $K_{2}{\mathrm{CO}}_{3}$ in MeOH provides a 1:1 mixture of compounds **68** and **69**, characterized by a fully conjugated B-ring, in an overall yield of 90%. Furthermore, compound **67** can be further elaborated to yield compounds **70** and **71**, corresponding to the ingenane- and rhamnofolane-type skeletons, respectively (Scheme 15).

## 3. Medicinal Chemistry of Lathyranes

Following the exploration of the chemical reactivity and bioinspired transformations of the lathyrane core discussed in the previous sections, these tricyclic diterpenoids have also attracted significant interest in medicinal chemistry due to their therapeutic potential. Their complex polycyclic framework, combined with a high level of oxygenation and multiple ester functionalities, offers a highly tunable and readily derivatizable three-dimensional scaffold. This structural versatility enables the fine modulation of key determinants of bioactivity, such as lipophilicity and the strategic positioning of aromatic moieties. Consequently, the lathyrane core represents an excellent platform and a privileged scaffold for the development of new bioactive compounds, bridging the chemical manipulation of the 5/11/3 tricyclic system with targeted pharmacological applications

### 3.1. P-Glycoprotein Inhibitors

Multidrug resistance (MDR) represents one of the major challenges in cancer chemotherapy, as tumors exhibiting resistance to a specific chemotherapeutic agent frequently develop cross-resistance to structurally and functionally unrelated drug classes. One of the primary mechanisms underlying MDR is the overexpression of ATP-binding cassette (ABC) transporters, which are membrane proteins mediated in the active efflux of xenobiotics. Among these, P-glycoprotein (P-gp, ABCB1) is the most extensively characterized, playing a pivotal role in reducing the intracellular accumulation of chemotherapeutic agents via an ATP-dependent extrusion mechanism [39,40,41]. Consequently, the development of potent P-gp inhibitors is a critical strategy to counteract MDR-related treatment failures, although clinical translation remains challenging due to the significant side effects and systemic toxicity of currently available reference inhibitors [42].

A milestone contribution to the development of novel natural product-based P-gp inhibitors was provided by Sousa et al., who established a quantitative structure–activity relationship (QSAR) framework for macrocyclic diterpenes as modulators of P-gp-mediated MDR [43]. By analyzing a dataset of more than 50 lathyrane- and jatrophane-type congeners, that study identified the key molecular determinants governing P-gp inhibition, representing one of the first systematic efforts to rationalize their activity. The QSAR analysis revealed that lipophilicity is the dominant driver of biological activity, strongly correlating with enhanced MDR-reversal effects, whereas an excessive number of polar functionalities negatively impacts performance. In parallel, descriptors related to charge distribution and molecular polarity highlighted the need for a delicate balance between hydrophobicity and electrostatic interactions to achieve optimal target engagement within the transporter cavity.

**Figure 4:**
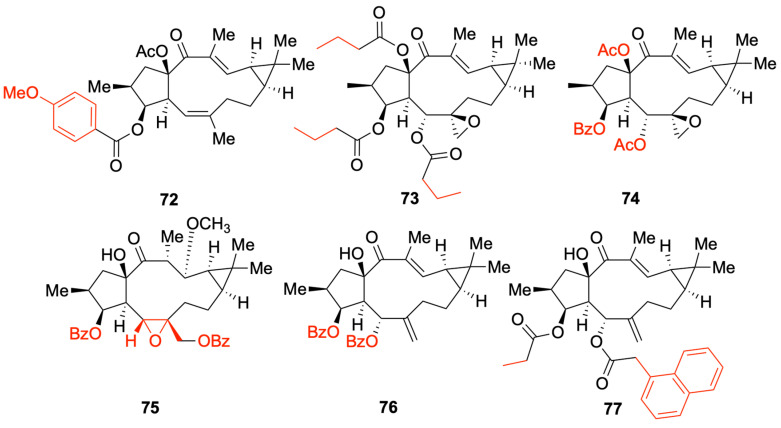
Structures of P-gp inhibitors **72**–**77**.

In this context, Ferreira and co-workers investigated a series of jolkinol D derivatives obtained through selective acylation at C3, identifying jolkinoate K (**72**) as one of the most potent P-gp modulators [44]. This compound exhibited remarkable MDR-reversal activity, with FAR values exceeding 100 at 20 μM, significantly outperforming the VPM. Biological assays further demonstrated that **72** restored drug sensitivity in a large fraction of resistant cells, reverting up to ≈95% of the MDR population to a chemosensitive phenotype in a dose-dependent manner. From a structural standpoint, aromatic acyl substituents at C3 play a key role in enhancing activity, likely by engaging in favorable hydrophobic and π–π stacking interactions within the P-gp drug-binding pocket. Conversely, non-aromatic or overly flexible aliphatic chains were generally less effective, underscoring the importance of both electronic and steric factors (Figure 4).

The same research group later investigated a series of epoxy-lathyrane metabolites isolated from the methanolic extract of *Euphorbia boetica* aerial parts, highlighting how the specific acylation pattern dictates biological activity [45]. Among the synthesized derivatives, epoxyboetirane J (**73**) emerged as the most promising agent, exhibiting strong P-gp inhibitory activity (FAR=83.7 at 20 μM), followed by epoxyboetirane M (**74**, FAR=80.6 at 20 μM), both outperforming VPM. Structure–activity relationship (SAR) analysis suggested that the enhanced activity of **73** is primarily driven by the presence of bulky alkanoyl substituents, which likely improve membrane permeability and promote more effective interactions within the large hydrophobic binding pocket of P-gp. Meanwhile, the performance of **74** supported the beneficial role of aromatic substituents, which enable efficient binding through localized π-interactions within the transporter (Figure 4).

In their continuing studies, in order to dissect the pharmacophore of lathyranes, Ferreira et al. identified methoxyboetirane B (**75**) as a highly promising chemo-sensitizing lead [46]. This compound, deriving from the rearrangement of the epoxylathyrol (**1**) framework, displayed remarkably high P-gp inhibitory activity, retaining potent modulation in combination with doxorubicin (at a 5:1 ratio) on ABCB1-transfected L5178Y cells even at submicromolar concentrations (${\mathrm{IC}}_{50}=0.057\pm 0.020$ μM). This enhanced potency is strictly related to its 3,17-diaromatic acylation pattern, which optimizes spatial interactions within the hydrophobic drug-binding pocket of the transporter through extended π–π stacking, while a favorable balance between lipophilicity and molecular size ensures efficient membrane permeation (Figure 4).

In another comprehensive SAR study focused on lathyrol derivatives, Jiao et al. identified **76** and **77** as potent modulators of ABCB1-mediated multidrug resistance [47]. Both compounds underscore the critical importance of aromatic substitution on the lathyrane core to enhance hydrophobic interactions within the large and flexible P-gp binding cavity. Notably, **77** further demonstrates how the fine-tuning of substituent size can optimize target engagement. Both compounds significantly outperformed the reference inhibitor VPM in reversing adriamycin resistance in MCF-7/ADR cells, with **76** achieving up to a 4.8-fold improvement over the control (Figure 4).

**Figure 5:**
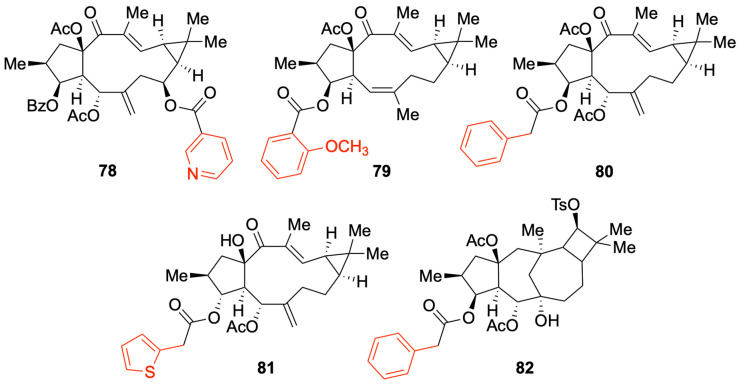
Structures of P-gp inhibitors **78**–**82**.

In a complementary approach, Wu et al. explored the use of biocatalysis employing the fungi *Mortierella ramanniana*, *Mucor circinelloides*, and the actinomycete *Nocardia iowensis* to access structurally novel lathyrane derivatives through regioselective hydroxylation at non-activated positions [48]. Although most hydroxylated metabolites exhibited a dramatic loss of direct cytotoxicity, the subsequent acylation of these novel hydroxy intermediates with lipophilic acyl chlorides led to a marked increase in chemo-sensitizing activity, as exemplified by compound **78** (FAR=4.9 at 20 μM). This outcome reinforces the central role of hydrophobic and aromatic interactions in governing the potency of lathyrane-based P-gp inhibitors (Figure 5).

Reis et al. investigated a series of jolkinol D-derived lathyranes as modulators of ABCB1-mediated multidrug resistance, identifying jolkinoate P (**79**) as a potent P-gp inhibitor, with FAR values reaching 33.3 at 2 μM [49]. Mechanistic insights indicated that **79** directly interacts with the transporter, exhibiting a concentration-dependent modulation of ATPase activity consistent with a dual role as both a slowly transported substrate and a competitive inhibitor. Moreover, in combination studies, it displayed a very strong synergism with doxorubicin (Combination Index, CI=0.09), effectively restoring drug sensitivity in resistant cancer cells. Overall, this compound exemplifies how the fine-tuning of aromatic substitution on the lathyrane scaffold can enhance activity through optimized hydrophobic and π–π interactions within the flexible P-gp binding cavity (Figure 5).

Liu and Cheng explored instead the chemical and microbial transformation (via *Mucor polymorphosporus* and *Cunninghamella elegans*) of euphorbia factor $L_{1}$ (**2**) to generate structurally diversified lathyrane derivatives, identifying the biotransformed congener **80** as the most active modulator of P-gp [50]. This derivative exhibited enhanced inhibitory activity compared to the parent compound, with an ${\mathrm{IC}}_{50}$ of 15.50 μM (versus 34.97 μM for 2). Mechanistic investigations revealed that **80** exerts its activity primarily through the down-regulation of P-gp expression at the protein level, rather than acting at the transcriptional level of the *MDR1* gene, highlighting a distinct mode of action compared to classical competitive efflux inhibitors (Figure 5).

Gao et al. investigated an alternative drug discovery strategy by synthesizing a small library of euphorbia factor $L_{3}$ (**17**) derivatives obtained through configuration inversion at C3 [51]. This approach enabled access to derivatives bearing the less common 3R configuration (H−3β), allowing a systematic evaluation of stereochemical effects on both antiproliferative and MDR-reversal activities. Biological screening revealed that several compounds displayed significant cytotoxicity, with derivative **81** emerging as the most potent antiproliferative agent, exhibiting an ${\mathrm{IC}}_{50}=1.3$ μM against HepG2 cells. Notably, the same compound emerged as the most effective MDR modulator when co-administered with paclitaxel in resistant MCF-7/Tax cells, achieving a reversal fold of up to 16.1 and outperforming the reference inhibitor VPM. These results suggest that configuration inversion can introduce novel biological features while fully preserving strong P-gp inhibitory properties (Figure 5).

In a recent study, Zhan et al. expanded the chemical space of lathyrane diterpenes through a combinatorial modification strategy targeting both the core scaffold and the ester side chains [52]. This approach enabled the generation of a large and structurally diverse library of derivatives, allowing a comprehensive assessment of the structural features governing MDR-reversal activity. Among the synthesized compounds, derivative **82** emerged as an exceptionally potent modulator of P-gp-mediated multidrug resistance, exhibiting an extraordinary reversal fold of 151.3 and an ${\mathrm{IC}}_{50}=0.54\pm 0.71$ μM on MCF-7/ADM cells, far exceeding previously reported analogues. This remarkable activity can be attributed to its favorable physicochemical profile, characterized by increased molecular weight, large molecular volume, and elevated lipophilicity (cLogP≈6.2), consistent with the general requirements for effective P-gp target engagement. SAR analysis of the library further highlighted the importance of key structural elements of the standard lathyrane core, including the presence and positioning of the double bonds (C6 = C17 and C12 = C13) and the nature of the ester substituents. Notably, the unusual 5/7/7/4 fused-ring skeleton of **82** suggests that significant rearrangements of the core scaffold are well tolerated and may even enhance the overall biological profile (Figure 5).

In light of these cumulative results, the lathyrane framework represents an excellent starting platform for the design of potent efflux pump modulators. To maximize binding affinity within the large transporter cavity, strategic functionalization of the diterpenoid core with aromatic ester groups is essential to ensure effective π–π stacking interactions, while carefully maintaining an appropriate lipophilic balance.

### 3.2. Treatment of Alzheimer’s Disease

Alzheimer’s Disease is a chronic neurodegenerative disorder and the most prevalent form of senile dementia [53]. While the exact etiology of AD has not been fully elucidated, oxidative stress has been implicated as a key driving factor in its pathogenesis. For this reason, the development of dual-action antioxidants and acetylcholinesterase (AChE) inhibitors has emerged as a promising therapeutic strategy. Several natural products exhibit potent antioxidant properties; among these, *Euphorbia* (Euphorbiaceae) diterpenes are well-known for their radical scavenging capacity [54], with specific myrsinane-type variants demonstrating remarkable neuroprotective effects [55].

**Figure 6:**
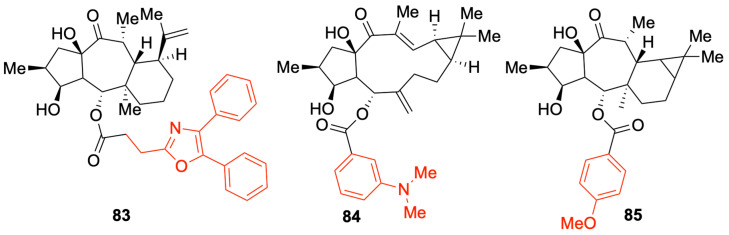
Structures of AChE inhibitors **83**–**85**.

Gao et al. described the semi-synthesis of a library of lathyrane-type derivatives starting from natural *Euphorbia* factors [56]. This synthetic campaign involved an elegant, multi-step biomimetic process combining a concise reductive olefin coupling with a visible-light-triggered, regioselective cyclopropane ring-opening. Among the synthesized compounds, the myrsinane derivative **83** displayed potent anti-AChE activity with an ${\mathrm{IC}}_{50}$ of 8.3 μM, outperforming the reference positive control (tacrine, ${\mathrm{IC}}_{50}=11.6$ μM) (Figure 6). The docking analysis highlighted how compound **83** forms a stable complex within the hydrophobic binding cavity of AChE. Specifically, the two aromatic rings of the derivative are engaged in key π-alkyl interactions with the hydrophobic side chains of ILE296 and LEU305, stabilizing the ligand-enzyme complex inside the binding pocket. In parallel, compound **83** displayed a remarkable neuroprotective effect by decreasing the rate of apoptosis in H_2_O_2_-treated SH-SY5Y neuroblastoma cells.

In a subsequent study, the same research group reported the generation of thirty-two premyrsinane and thirty-two lathyrane derivatives, derived from the naturally abundant euphorbia factor $L_{3}$ (**17**) through a multi-step chemical approach utilizing bioinspired skeletal conversion strategies [57]. The cholinesterase inhibitory and neuroprotective properties of these derivatives were systematically examined to assess the therapeutic potential of both diterpene classes. The biological screening revealed that the lathyrane core is a crucial determinant for strong AChE inhibition. Interestingly, SAR data indicated that proper esterification at the lathyrane C5 position enhances anti-AChE potency, whereas acylation at the C3 position significantly reduces the effect. Among the evaluated library, compound **84**, bearing a 3-dimethylaminobenzoyl moiety at C5, demonstrated the most potent anti-AChE activity with an ${\mathrm{IC}}_{50}$ of 7.1 μM, a value 1.5-fold lower than that of tacrine. However, despite their good anti-AChE activity, lathyrane derivatives generally exhibited neurotoxicity toward SH-SY5Y cells. Conversely, the related derivative 85 showed remarkable neuroprotective effects in cell-based assays, maintaining a cell viability rate of 113.5% at 12.5 μM against oxidative damage, more than doubling the 51.2% survival rate observed in the vehicle-treated model group (Figure 6). The mechanism of the neuroprotective activity of premyrsinane derivative **85** was demonstrated through immunofluorescence and Western blot analysis, confirming its ability to inhibit H_2_O_2_-induced cellular apoptosis.

The comprehensive SAR analysis of these three related classes of diterpenes revealed distinct, scaffold-dependent pharmacological profiles. Specifically, the lathyrane skeleton serves as an effective starting point for the development of potent anti-AChE compounds, whereas the premyrsinane framework exhibits optimized neuroprotective activity. On the other hand, the myrsinane core successfully integrates both pharmacological traits; indeed, compound **83** proved to be simultaneously an excellent AChE inhibitor and a highly effective neuroprotective agent.

### 3.3. Anticholestatic Derivatives

Cholestasis is a pathological condition characterized by impaired bile flow, which leads to the toxic accumulation of bile acids (BAs) in the liver. As the disease progresses, it induces severe hepatocellular damage and may evolve into chronic conditions such as PBC and PSC [58]. The primary therapeutic objective is to eliminate excess BAs or restore physiological bile flow. Currently, UDCA and OCA are the only FDA-approved drugs for cholestasis treatment; however, only 50–60% of patients respond adequately to these therapies, leaving a significant clinical gap.

The PXR is a ligand-activated nuclear receptor that regulates the intricate metabolic networks governing xenobiotic and endogenous steroid clearance. Accumulating evidence highlights the pivotal role of PXR signaling in maintaining BA homeostasis. Upon activation, PXR promotes BA detoxification by up-regulating phase I hydroxylation and conjugation pathways. Specifically, it induces the expression of cytochrome P450 enzymes, such as CYP3A and CYP2B, which convert hydrophobic BAs into hydrophilic hydroxylated derivatives, facilitating their subsequent conjugation. Concurrently, PXR activation upregulates the expression of P-glycoprotein (P-gp, encoded by *MDR1*), which actively transports the detoxified BA metabolites across the canalicular membrane into the bile or urine for excretion. Consequently, hPXR has emerged as a highly promising therapeutic target for anticholestatic drug development.

**Figure 7:**
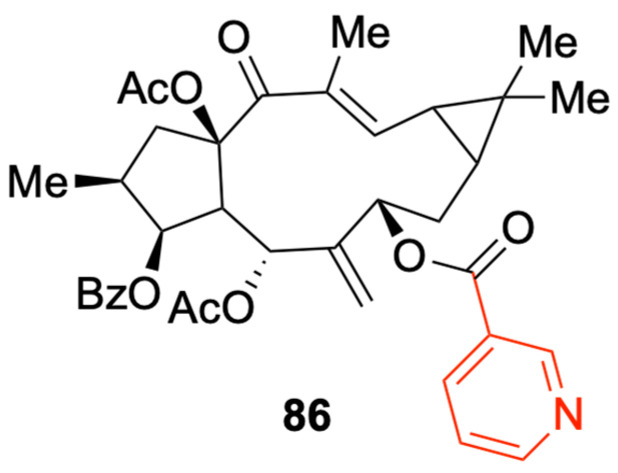
Structure of anticholestatic derivative **86**.

In this context, Huang et al. generated a library of hPXR agonists through the structural modification of lathyrane diterpenoids obtained via bioassay-guided isolation from *Euphorbia lathyris* [59]. SAR analysis and molecular docking simulations indicated that the acyloxy group at C7 and the C14 carbonyl functionality are essential determinants for robust receptor binding. Among the synthesized library, compound **86** emerged as the most potent agonist, eliciting a remarkable 6.9-fold increase in hPXR reporter gene activity and significantly up-regulating the transcriptional expression of the downstream target genes *CYP3A4*, *CYP2B6*, and *MDR1*. Notably, the presence of a 7-nicotinoyloxy moiety is pivotal for potency, as its substitution with alternative acyl groups, such as a benzoyl moiety, is highly detrimental to receptor activation (Figure 7). To better understand the role of this nicotinic scaffold within the binding pocket, docking analysis on hPXR was performed. The results indicated that a combination of π–π stacking interactions alongside a key hydrogen bond between the aromatic nitrogen of the pyridine ring and TRP299 is fundamental for the high biological activity of this derivative.

### 3.4. Anti-Inflammatory Lathyrane Diterpenoids

Inflammation is a protective immune response triggered by tissue trauma, cellular stress, or pathogen invasion. However, severe, uncontrolled, or chronic systemic acute inflammation can drive severe tissue damage, organ failure, and systemic pathologies. Current clinical options for managing inflammatory disorders rely primarily on anti-inflammatory steroids, NSAIDs, COX-2 inhibitors, and targeted biological agents. Natural products, particularly terpenoids, have gained widespread recognition as privileged structures with remarkable immunomodulatory profiles. Specifically, certain lathyrane diterpenoids have demonstrated a potent capacity to inhibit NO production, subsequently reducing downstream inflammatory cytokines. This effect is achieved through the down-regulation of iNOS and NF-κB expression, alongside the inhibition of IκBα phosphorylation [60].

**Figure 8:**
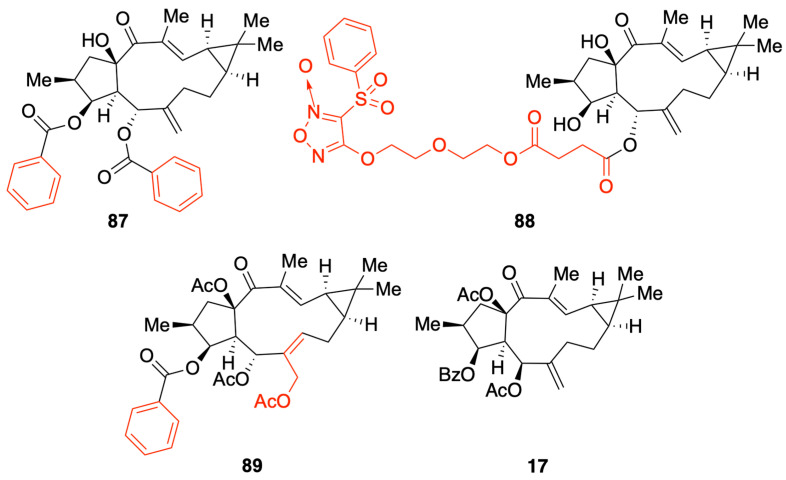
Structures of anti-inflammatory derivatives **87**–**89** and **17**.

In this context, Wang et al. synthesized two series of euphorbia factor $L_{3}$ (**17**) derivatives esterified with various fatty and aromatic acids [61]. Among the prepared library, compound **87** exhibited the most potent inhibitory profile on NO production, displaying an ${\mathrm{IC}}_{50}$ value of 3.38±1.03 μM without inducing systemic cytotoxicity. Preliminary SAR studies revealed that the anti-inflammatory activity of the lathyrol scaffold is highly dependent on the presence of a free hydroxyl group at the C7 position, whereas esterification of the C5 hydroxyl group with aromatic acids significantly enhances its overall anti-inflammatory potency (Figure 8).

In their continuing optimization of the lathyrane core, the same research group subsequently reported three series of epoxylathyrol and lathyrol derivatives incorporating heterocyclic architectures, specifically pyrazole, thiazole, and furoxan rings [62]. Pharmacological evaluation identified compound **88** as an exceptional agent, capable of strikingly suppressing lipopolysaccharide-induced NO production in RAW264.7 macrophages with a submicromolar potency (${\mathrm{IC}}_{50}=0.38\pm 0.18$ μM). Mechanistic analysis indicated that the phenylsulfonyl-substituted furoxan moiety is a pivotal pharmacophoric element to enhance the anti-inflammatory properties of the lathyrane core. Furthermore, compound **88** significantly attenuated intracellular reactive oxygen species levels, a protective effect tightly correlated with the regulation and modulation of the Nrf2/HO-1 signaling pathway (Figure 8).

Beyond general inflammation, the efficacy of classical anti-inflammatory agents—such as NSAIDs, corticosteroids, and colchicine—has been extensively evaluated for the management of metabolic-driven immune conditions such as gout. Gout is a painful autoinflammatory disease triggered by the deposition of monosodium urate crystals, causing debilitating acute arthritis and long-term multisystemic damage, including cardiovascular and renal complications. Zhuang et al. provided the first report on the application of synthetic lathyrane-based diterpenoids for the treatment of gouty arthritis [63]. They observed that euphorbia factor $L_{3}$ (**17**) and its derivative **89**, both possessing an intact acyloxy group at C15 alongside hydrophobic aromatic architectures at C3 and C5, strikingly inhibited IL-1β production by blocking inflammasome activation. When evaluated in vivo, the intraperitoneal administration of euphorbia factor $L_{3}$ (**17**) as a model compound in a C57BL/6 mouse model of acute gouty paw edema demonstrated a significant anti-inflammatory and tissue-protective effect, validating the therapeutic potential of this scaffold (Figure 8).

In short, these findings demonstrate that the anti-inflammatory activity of the lathyrane framework is strictly dependent on specific functionalization patterns. While a free hydroxyl group at C7 is required to maintain core activity, the introduction of hydrophobic aromatic esters at C5 and C3—or the preservation of the acyloxy group at C15—is essential to optimize target engagement. These structural modifications dictate the selectivity of the scaffold, allowing it to suppress general inflammatory mediators like NO via NF-κB modulation, or to block specific autoinflammatory pathways such as inflammasome activation in gouty arthritis.

### 3.5. Diterpenoids-Based HIV Inhibitors

Recent studies have increasingly highlighted the potential of *Euphorbia*-derived diterpenoids and their semisynthetic derivatives as promising leads for anti-HIV therapy. AIDS, a severe condition caused by HIV, profoundly compromises the host immune system, leading to a progressive loss of immune function that has resulted in approximately 40 million deaths globally since its emergence [64,65].

**Figure 9:**
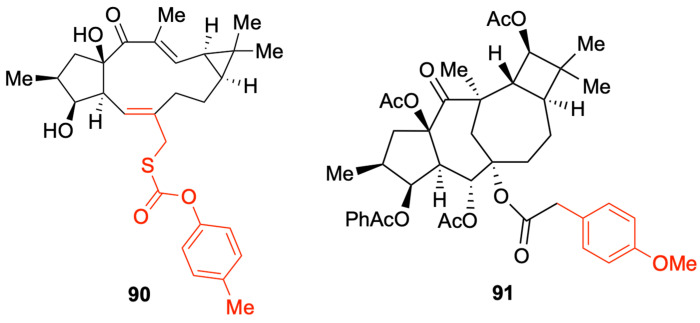
Structures of anti-HIV derivatives **90** and **91**.

In the search for novel natural product-based anti-HIV agents, Gao and co-workers exploited the abundant lathyrane-type diterpene, euphorbia factor $L_{3}$ (**17**), as a versatile starting material [66]. The lathyrane core was functionalized by leveraging a [3,3]-sigmatropic rearrangement to successfully introduce a thioformate group at the C17 position. The incorporation of this sulfur atom into the terpenoid architecture significantly optimized its pharmacological properties, improving both binding affinity and metabolic stability. Among the synthesized library, the sulfur-containing lathyrane derivative **90**, bearing an O-(p-tolyl) carbonothioate moiety at C17, exhibited the most potent anti-HIV profile, displaying half-maximal effective concentration (${\mathrm{EC}}_{50}$) values of 11.3 μM against HIV-1 NL 4.3 and 6.6 μM against HIV-2 ROD (Figure 9).

In a subsequent study, Gao’s research group expanded their structural modifications to include three naturally rare classes of rearranged *Euphorbia* diterpenoids (specifically 5/7/7/4 eupholathones, 6/11/3 lathyranones, and 5/11 integerrimenes) accessed via a biomimetic skeletal conversion strategy [67]. Among these structurally diverse frameworks, compounds possessing the eupholathone-type skeleton exhibited the most compelling antiviral activity. SAR analysis revealed that the free hydroxyl group at position 6 of the eupholathone core is a pivotal determinant for enhancing anti-HIV potency. Furthermore, functionalization with aromatic esters proved to be particularly advantageous. Notably, the eupholathone aromatic ester **91** demonstrated the most effective anti-HIV-1 profile, exhibiting a remarkable ${\mathrm{EC}}_{50}$ value of 1.51 μM and a selectivity index greater than 66.2 (Figure 9).

In these studies, Gao’s group took advantage of the lathyrane skeleton to functionalize and modify its structure, exploring an extensive library of compounds. Their findings reveal that the rearranged eupholathone skeleton represents an excellent starting point for the development of new compounds with anti-HIV activity. Notably, the lead compound **91** demonstrated high potency against the virus while showing no cytotoxicity in cell-based assays.

### 3.6. Antifungal Derivatives

Over the past few decades, the incidence of systemic fungal infections has increased significantly, primarily driven by the expanding population of immunocompromised patients and the widespread clinical use of corticosteroids and immunosuppressive therapies. The most prevalent opportunistic fungal pathogens belong to the *Cryptococcus*, *Aspergillus*, *Candida*, and *Pneumocystis* genera. In clinical settings, triazole antifungal agents serve as the primary first-line defense for treating systemic mycoses. Among these, fluconazole is one of the most widely prescribed therapeutics owing to its excellent oral bioavailability, high efficacy, and favorable safety profile. However, the extensive and prolonged use of azoles in recent years has led to the rapid emergence of secondary resistance. In *Candida albicans*, azole resistance is mediated by multiple molecular mechanisms, among which the overexpression of ATP-binding cassette (ABC) efflux pumps—which actively extrude antifungal agents from the intracellular space—plays a dominant role [68].

**Figure 10:**
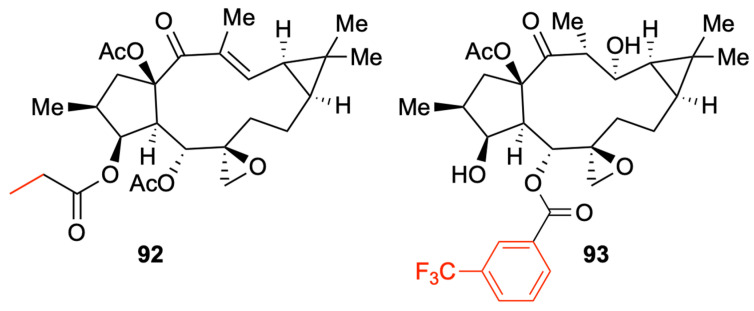
Structures of antifungal derivatives **92** and **93**.

Certain diterpenes isolated from the roots of *Euphorbia lathyris* have been reported to exhibit noteworthy antifungal properties [69]. Given that macrocyclic lathyrane and jatrophane diterpenoids derived from *Euphorbia* species have established profiles as potent modulators of the human ABCB1 (P-gp) efflux pump in multidrug-resistant cancer cells [70], they represent ideal candidates to target fungal counterparts. Concurrently, Ferreira and co-workers evaluated the capacity of a library of lathyrol and epoxylathyrol derivatives to inhibit the Cdr1p and Mdr1p efflux pumps of *C. albicans*, utilizing a *Saccharomyces cerevisiae* heterologous expression model to decipher potential resistance-reversing mechanisms [71]. In chemo-sensitization transport assays, remarkable synergistic combinations were observed for epoxyboetirane K (**92**) and euphoboetirane N (**93**) when co-administered with fluconazole in the AD-*CDR1* yeast strain, which exclusively overexpresses the major Cdr1p transporter. Notably, the addition of these chemosensitizers reduced the effective inhibitory concentration of fluconazole by 23- and 52-fold, respectively. From a structural perspective, the highly potent derivative **93** incorporates a 3-trifluoromethylbenzoyl moiety at the C3 position and is structurally characterized by the absence of the typical endocyclic macrocyclic double bond, alongside the presence of an additional hydroxyl functionality at C13 (Figure 10).

### 3.7. PROTACs

PROTACs have recently emerged as a revolutionary therapeutic strategy for the selective degradation of specific protein targets by hijacking the endogenous UPS [72]. Unlike classical small-molecule inhibitors, PROTACs operate via a sub-stoichiometric catalytic mechanism and do not require high-affinity binding to an active site to exert their activity, opening up exciting avenues in drug discovery. In this context, their application as chemical biology tools for target identification (*target ID*) has generated significant interest; a prime example is the recent elucidation of previously unrecognized non-kinase targets of the multi-kinase inhibitor sorafenib using PROTAC technology [73]. These breakthroughs have encouraged researchers to explore the potential of PROTACs to unravel the elusive molecular targets of complex natural products, including lathyrane diterpenoids.

**Figure 11:**
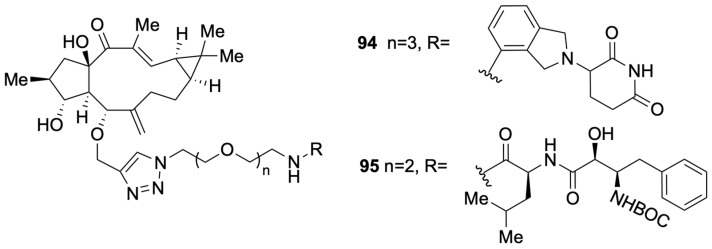
Structures of PROTAC derivatives **94** and **95**.

In this framework, an elegant chemical biology strategy based on PROTAC technology was recently developed by Chen et al. to identify the primary biological target of these macrocycles [74]. By designing a lathyrol-derived PROTAC (**94**), the researchers successfully exploited targeted protein degradation as a functional readout, leading to the discovery of MAFF as a primary cellular target of lathyranes. Mechanistically, **94** modulates MAFF dimerization dynamics, selectively inhibiting the formation of transcriptionally repressive MAFF homodimers while promoting the assembly of active MAFF–Nrf2 heterodimers. This transcriptional shift triggers the robust activation of the Nrf2/HO-1 signaling axis, ultimately driving pronounced antioxidant and anti-inflammatory cellular effects [74]. These findings not only provide a definitive molecular rationale for the anti-inflammatory activity traditionally associated with lathyrane diterpenoids but also underscore the versatility of their polyoxygenated scaffold in engaging complex PPI networks, highlighting a previously unrecognized link between this class of natural products and the regulation of oxidative stress pathways (Figure 11).

The same research group subsequently focused on the design of a second generation of lathyrol-based PROTACs to optimize their anti-inflammatory potential [75]. A structurally diversified library was synthesized by systematically varying both the nature of the E3 ligase ligand (evaluating CRBN, VHL, cIAP, and MDM2 recruiters) and the chemical composition and length of the spacer linker, while preserving the lathyrol core as the warhead (POI-binder). Among the synthesized chimeras, compound **95** emerged as the most potent analogue, displaying improved inhibition of LPS-induced NO production (${\mathrm{IC}}_{50}\approx 5$ μM) alongside a low cytotoxicity profile, while fully retaining the capacity to induce MAFF degradation in a time- and concentration-dependent manner.

Further mechanistic investigations indicated that **95** exerts its biological effects through the coordinated modulation of key inflammatory and oxidative stress pathways. It efficiently activates the Keap1/Nrf2 signaling axis while concurrently suppressing NF-κB signaling by inhibiting both its protein expression and nuclear translocation. Moreover, **95** promotes the up-regulation of autophagy-related pathways, suggesting a multifaceted mode of action that synergistically integrates redox regulation, anti-inflammatory signaling, and cellular homeostasis.

Overall, these milestones demonstrate how the lathyrane framework is uniquely suited for PROTAC-based enhancement. Targeted protein degradation opens exciting new paths, not only for target identification but also for the strategic fine-tuning of the biological activity of complex natural products. However, despite these promising results, SARs regarding linker length and ligase recruitment remain preliminary, and further structural optimization is required to fully mature lathyrol-based PROTACs into drug-like clinical entities.

## 4. Conclusions

This review summarizes approximately five decades of intensive research into the chemistry and therapeutic potential of lathyrane diterpenoids. Although these complex secondary metabolites were first identified over a century ago, they have more recently captured the attention of the organic and medicinal chemistry communities due to their unique carbon frameworks and compelling pharmacological profiles. The inherent conformational rigidity and dense functionalization of the 5/11/3 tricyclic scaffold have enabled the discovery of novel transannular rearrangements, inspiring elegant biomimetic strategies to access rare and complex secondary metabolite architectures under laboratory conditions.

In medicinal chemistry, lathyrane diterpenoids have established themselves as scaffolds capable of interacting with multiple biological targets: they exhibit a broad spectrum of bioactivities, including cytotoxic, anti-inflammatory, neuroprotective, and antiviral effects, alongside their well-characterized capacity to reverse MDR by inhibiting ABC efflux pumps across different kingdoms. Furthermore, cutting-edge applications, such as the development of lathyrol-based PROTACs, have recently demonstrated the versatility of this core, opening new avenues for precise target identification—such as the MAFF protein—and the fine-tuning of biological efficacy through targeted protein degradation.

To date, a significant number of members of this class of diterpenes have been isolated and characterized, with new congeners disclosed annually, underscoring that the chemical space of the *Euphorbiaceae* family is far from exhausted. Looking ahead, the integration of synthetic methodology, medicinal chemistry, and advanced pharmacology will be essential to fully unlock the therapeutic potential of these metabolites. In particular, a deeper, structurally guided understanding of their macromolecular target engagements will drive the rational design of simplified synthetic analogues with improved physicochemical properties, enhanced metabolic stability, and minimized toxicity. Overall, lathyrane diterpenoids represent a dynamic and expanding field at the interface of natural product chemistry and drug discovery, destined to deliver both fundamental scientific insights and concrete therapeutic opportunities in the near future.

## Data Availability

No new data were created or analyzed in this study. Data sharing is not applicable to this article.

## Abbreviations

The following abbreviations are used in this manuscript:

GGPP	Geranylgeranyl pyrophosphate
MDR	Multidrug resistance
NPC	Neural Progenitor Cells
AN	Acetonitrile
PTSA	*p*-Toluensulfonic acid
SET	Single electron transfer
FAR	Fluorescence activity ratio
VPR	Reference modulator verapamil
AD	Alzheimer’s Disease
Bas	Bile acids
UDCA	Ursodeoxycholic acid
OCA	Obeticholic acid
ABC	ATP-binding cassette
QSAR	Quantitative structure–activity relationship
SAR	Structure-activity relationship
PBC	Primary biliary cholangitis
PSC	Primary sclerosing cholangitis
PXR	Pregnane X receptor
NSAIDs	Non-steroidal anti-inflammatory drugs
AIDS	Acquired Immune Deficiency Syndrome
HIV	Human immunodeficiency virus
PROTACs	Proteolysis Targeting Chimeras
MAFF	V-maf musculoaponeurotic fibrosarcoma oncogene homolog F
PPI	Protein–protein interaction
